# Differences in PI_total_ of *Quercus liaotungensis* seedlings between provenance

**DOI:** 10.1038/s41598-021-02941-5

**Published:** 2021-12-06

**Authors:** Xiangchun Hao, Shuai Zhou, Lijun Han, Yu Zhai

**Affiliations:** Shanxi Academy of Forestry and Grassland Sciences, Taiyuan, China

**Keywords:** Ecology, Plant sciences

## Abstract

The performance index of overall photochemistry (PI_total_) is widely used in photosynthesis research, but the PI_total_ interspecies differences are unclear. To this end, seeds of *Quercus liaotungensis* from 10 geographical provenances were planted in two different climate types. Two years later, leaf relative chlorophyll content (SPAD) and chlorophyll *a* fluorescence transient of seedlings were measured. Meanwhile, the environmental factors of provenance location, including temperature, precipitation, solar radiation, wind speed, transpiration pressure, and soil properties, were retrieved to analyze the trends of PI_total_ among geographic provenance. The results showed that, in each climate type, there was no significant difference in SPAD and electron transfer status between PSII and PSI, but PI_total_ was significantly different among geographic provenances. The major internal causes of PI_total_ interspecies differences were the efficiency of electronic transfer to final PSI acceptor and the number of active reaction centers per leaf cross-section. The main external causes of PI_total_ interspecies differences were precipitation of the warmest quarter, solar radiation intensity in July, and annual precipitation of provenance location. PI_total_ had the highest correlation with precipitation of the warmest quarter of origin and could be fitted by the Sine function. The peak location and fluctuating trend of precipitation—PI_total_ fitted curve were different in two climate types, largely due to the difference of precipitation and upper soil conductivity in the two test sites. Utilizing the interspecific variation and trends of PI_total_ might be a good strategy to screen high and stable photosynthetic efficiency of *Q. liaotungensis* provenance.

## Introduction

*Quercus liaotungensis* is an essential deciduous tree in warm-temperate deciduous broad-leaved forests. It is a suitable species for establishing mixed broadleaf-conifer forests in the middle and low elevations of North China^1^. The regeneration of the *Q. liaotungensis* forest depends on sprouting. However, the growth of sprout seedling degraded, and the soil seed bank of *Q. liaotungensis* was lost due to predation, insect moth, and physiological death^2,3^. Therefore, the regeneration and establishment of *Q. liaotungensis* forest mainly rely on artificial breeding, so provenance selection is essential. Provenance selection will help to discover excellent germplasm resources of *Q. liaotungensis* for regeneration and establishment. More recent attention has focused on providing resource distribution, phenotypic traits, and the economic value of *Q. liaotungensis* seeds from different provenances^4,5^. A key aspect of sustainable forestry is to cultivate plants with efficient photosynthetic system^6,7^. Photosystem II (PSII) is driven by light energy and provides assimilation power to synthesize plant carbohydrates^8^. It is generally believed that PSII, an important component of the photosynthetic system, is most sensitive to environmental changes^9,10^. Currently, there is a lack of work for evaluating differences in the PSII activity between *Q. liaotungensis* provenances.

Chlorophyll *a* fluorescence is a potential and simple tool to analyze the performance of Photosystem II (PSII). This technology is able to discriminate differences due to the influence of environmental factors on the photochemical activity of PSII, such as temperature^11,12^, water^13,14^, salinity^15,16^, light^17,18^, and insect feeding^19,20^. Different parameters have been used to describe the photochemical activity of PSII. Butler and Kitajima first constructed the maximum quantum yield of primary PSII photochemistry (*F*_v_/*F*_m_) based on the characteristics of the OJIP curve in 1975^21^. However, this parameter was shown to be nonspecific^22^ and often insensitive^14^. In 1999, Strasser et al. developed performance index of overall photochemistry (PI_ABS_) by using three independent OJIP curve parameters (*φ*_Po_—maximum quantum yield of primary PSII photochemistry, *ψ*_Eo_—efficiency with which a PSII trapped electron is transferred from ${\text{Q}}_{\text{A}}^{-}$ to PQ: and RC/ABS—the density of PSII reaction centers)^23^. PI_ABS_ could reflect the state of plant photosynthetic apparatus more accurately than *F*_v_/*F*_m_^24^, whereas PI_ABS_ was related only to the electron transport to the PQ pool^25^. Tsimilli-Michael and Strasser proposed the performance index of overall photochemistry (PI_total_)^26^. PI_total_ calculates by PI_ABS_ and *δ*_Ro_ (the efficiency of the electron from PQH_2_ is transferred to final PSI acceptors), which can fully describe the photochemical activity of the linear photosynthetic electron transfer chain^25^. Data from numerous studies indicated that PI_total_ decreased significantly in response to high PAR dose, high ambient temperature, low soil water content, K^+^-deficiency stress, Mg-deficiency stress, shade stress, and heat stress^27–31^. Simultaneously, PI_total_ increased significantly during the light-induced plasticity of plant growth^32^ and was considered the most salinity-sensitive parameter^33,34^. Widespread plant species often show extensive variation in morphological and growth characteristics as well as the substantial difference in stress resistance due to different individual selection pressures^35^. Existing studies have shown significant differences in the PSII photochemical activity from different provenances^36,37^. However, the mechanism and the trend of change for the interspecies differences of PSII photochemical activity are still unclear. Investigating PSII interspecies differences can help implement the OJIP-test to provenance trials, and obtain excellent germplasm resources with high and stable photosynthetic inorganic carbon assimilation ability.

In this paper, 2-year-old *Q. liaotungensis* seedlings coming from 10 different provenances were used as test materials. The difference in PI_total_ between provenances from internal factors (φ_Po_, ψ_Eo_, δ_Ro_, and RC/ABS) and external factors (temperature, precipitation, solar radiation, wind speed, transpiration pressure, and soil properties) in provenances was analyzed. Several studies have investigated that dry climate threatened *Q*. *liaotungensis* forest growth^38,39^. In order to verify the experimental results, two separate experiments were conducted in semi-arid and sub-humid distribution zones of *Q*. *liaotungensis*. We tested three different hypotheses: (1) There are differences among the *Q. liaotungensis* provenances of PI_total_. (2) These changes can be explained by the parameters used to calculate PI_total_ and the environmental factors of provenance. (3) The difference trend may be predicted. Our expected results were to 1) evaluate the degree of PSII interspecific difference of *Q. liaotungensis*. (2) Understand the patterns of variation observed.

## Materials and methods

### Test sites

The test was carried out simultaneously in Yangqu and Sanjiao test sites. The Yangqu test site is located in Yangqu County, Shanxi, China (38.0981° N, 112.7346° E, and 961 m above sea level), is a warm temperate semi-arid continental monsoon climate. The annual mean temperature is 8.5 °C, the annual rainfall is 430.4 mm, the annual average frost-free period is 164 days. The Sanjiao test site is located in Fushan County, Shanxi, China (35.9649° N, 112.0766° E, and 1197 m above sea level), is a warm temperate semi-humid continental monsoon climate. The annual mean temperature is 9.1 °C, the annual rainfall is 569.6 mm, the annual average frost-free period is 191 days. The soil type of the two test sites is loam.

### Test materials

**Figure 1:**
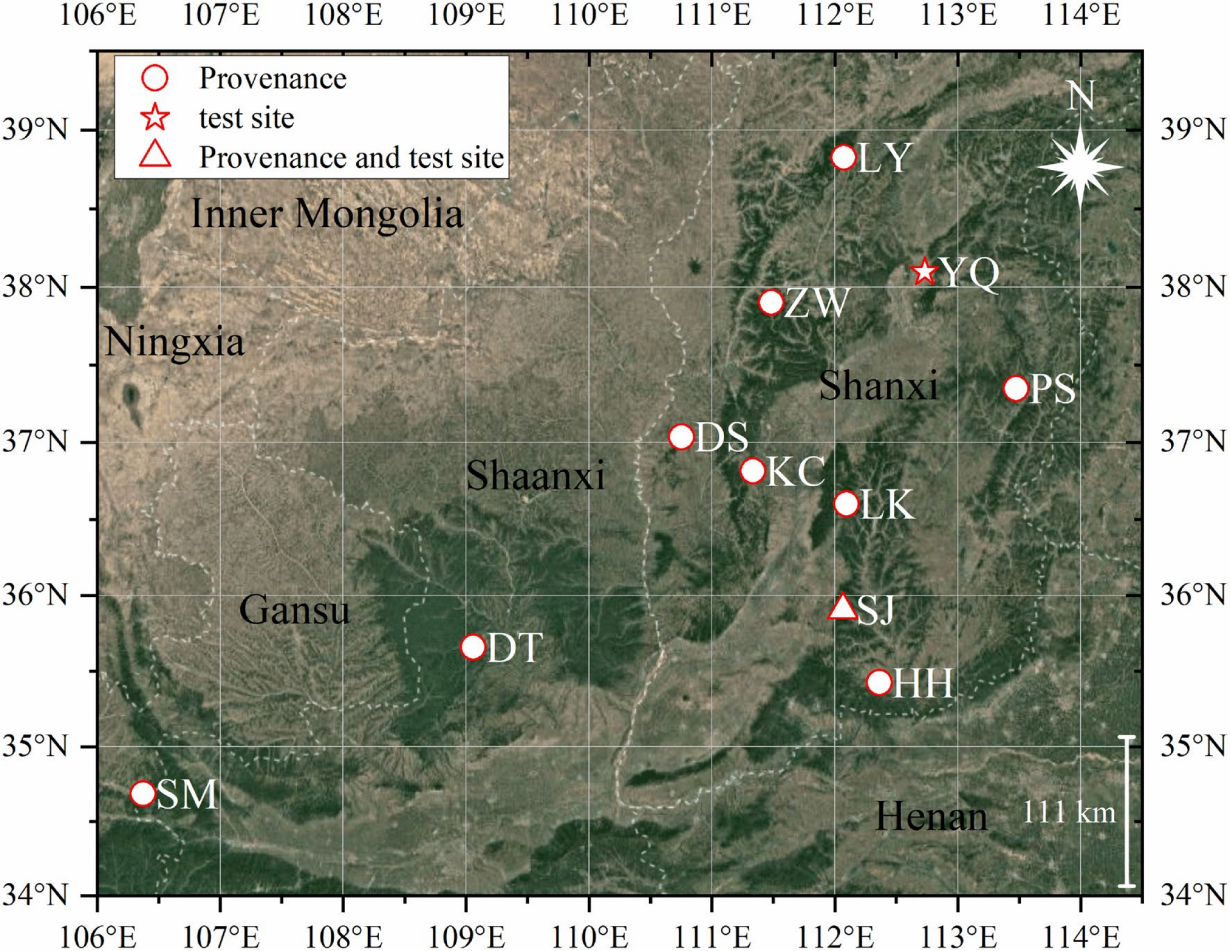
Geographical locations of *Quercus liaotungensis* provenances and test sites. The map is created using the Google Map Import plug-in of Origin 2018 (https://www.originlab.com/fileExchange/details.aspx?fid=344). Coordinate reference system is WGS-84. *PS* Pingsong provenance, *LY* Luyashan provenance, *KC* Kangcheng provenance, *HH* Henghe provenance, *LK* Lingkongshan provenance, *DS* Dongshan provenance, *SJ* Sanjiao provenance (and Sanjiao test site), *SM* Shanmen provenance, *ZW* Zhenwushan provenance, *DT* Diantou provenance, *YQ* Yangqu test site.

In the autumn of 2017, the seeds of *Q. liaotungensis* were collected from 10 provenances in the species' natural distribution range. The names, geographical locations, climate conditions, and soil types of all provenances were summarized in Fig. 1 and Table 1. For each provenance, seeds were collected from plus trees with plant spacing was greater than 50 m in the middle-aged forests. The fully mixed seeds were used as the provenance seed. Malathion was used to kill insects in seeds. The seeds were sown in the field in the autumn of the same year. The experiment had a randomized block design with three replications. At least 8000 seeds per replication were sown, and each seed was sowed at a distance of about 20 cm. Field management followed the normal agricultural practices in the two test sites. Two years later, vigorous seedlings from germinated seeds were evaluated for relative chlorophyll content (SPAD) and chlorophyll *a* fluorescence. All parameters were measured in August 2019.

**Table 1:** The names, geographical locations, climate conditions, and soil types of *Quercus liaotungensis* provenances.

Code	Longitude (E)	Latitude (N)	Altitude/m	Aver. temp. (℃)	precipitation (mm)	Soil type
PS	113.474°	37.348°	~ 1450	6.9	560.0	Loam
LY	112.074°	38.826°	~ 1960	2.8	490.5	Loam
KC	111.334°	36.814°	~ 1230	8.3	504.6	Loam
HH	112.362°	35.426°	~ 780	11.3	586.8	Loam
LK	112.095°	36.553°	~ 1530	6.7	565.2	Loam
DS	110.753°	37.035°	~ 1100	9.4	492.9	Loam
SJ	112.066°	35.907°	~ 1180	9.1	569.6	Loam
SM	106.374°	34.685°	~ 1770	7.4	630.6	Loam
ZW	111.484°	37.903°	~ 1960	4.0	507.6	Sandy loam
DT	109.059°	35.659°	~ 1100	9.2	544.5	Loam

*PS* Pingsong provenance, *LY* Luyashan provenance, *KC* Kangcheng provenance, *HH* Henghe provenance, *LK* Lingkongshan provenance, *DS* Dongshan provenance, *SJ* Sanjiao provenance, *SM* Shanmen provenance, *ZW* Zhenwushan provenance, *DT* Diantou provenance.

### Parameter measurement methods

The mature fully expanded and unshaded leaves of 30 vigorous seedlings from each provenance were used to monitor the chlorophyll *a* fluorescence transient and SPAD. SPAD and chlorophyll *a* fluorescence transient were sequentially measured on the same leaf. One measurement per seedling was taken, resulting in 30 measurements per provenance.

The SPAD value of leaf was measured with a chlorophyll meter (SPAD-502Plus, KONICA MINOLTA, Japan) and chlorophyll *a* fluorescence transient was measured with a PAM-fluorometer (FluorPen FP110, Photon Systems Instruments, Czech Republic). The chlorophyll *a* fluorescence transient measurement was made from leaves that were dark adapted for 20 min using leaf clips. The mesophyll was illuminated with saturated blue light (2 100 μmol m^−2^ s^−1^) for 1 s, and the fluorescence signals at intervals of 10 μs (before 600 μs), 100 μs (between 600 μs and 15 ms), 1 ms (between 15 and 100 ms), and 10 ms (after 100 ms) were recorded. The OJIP-derived parameters (Table 2) were calculated with reference to Stirbet et al. ^25^ and Holland et al. ^40^.

**Table 2:** The OJIP-derived parameters.

Parameter	Calculation	Description
F_O_		Minimum fluorescence
F_m_		Maximum fluorescence
M_0_	≈ 4 × (F_0.3 ms_ − F_0.05 ms_)/(F_m_ − F_O_)	Initial slope (in ms^–1^) of the O-J fluorescence rise
V_J_	= (F_2ms_ − F_O_)/ (F_m_ − F_O_)	Relative variable fluorescence at 2 ms
V_I_	= (F_30ms_ − F_O_)/ (F_m_ − F_O_)	Relative variable fluorescence at 30 ms
ψ_Ro_	= 1 − V_I_	Efficiency with which a PSII trapped electron is transferred to final PSI acceptors
RC/ABS	= [(F_m_ − F_O_)/F_m_]/(M_0_/V_J_)	The density of PSII reaction centers
φ_Po_	= (F_m_ − F_O_)/F_m_	Maximum quantum yield of primary PSII photochemistry
ψ_Eo_	= 1 − V_J_	Efficiency with which a PSII trapped electron is transferred from Q − A to PQ
δ_Ro_	= ψ_Ro_/ψ_Eo_	Efficiency with which an electron from PQH_2_ is transferred to final PSI acceptors
PI_total_	= (RC/ABS) × [φ_Po_/(1 – φ_Po_)] × ψ_Eo_/(1 − ψ_Eo_)] × [δ_Ro_/(1 − δ_Ro_)]	Performance index of overall photochemistry

Environmental factors (11 temperature factors, 8 precipitation factors, 12 monthly solar radiation intensity factors, 12 monthly average wind speed factors, and 12 monthly transpiration pressure factors) were retrieved from the WorldClim database (https://www.worldclim.org/data/worldclim21.html) with a resolution of 10 arc-minutes based on the geographical location of the provenance. 32 soil factors were retrieved from the Harmonized World Soil Database (HWSD v1.2) provided by the National Tibetan Plateau Data Center. Eliminating collinearity was executed when the Pearson correlation coefficient between environmental factors was greater than 0.9. 22 factors were finally retained (Table 3).

**Table 3:** Environmental factors for the geographical location of *Quercus liaotungensis* provenances after eliminating collinearity.

Factor	Units	Code
Annual mean temperature	℃	B1
Mean diurnal range	℃	B2
Annual precipitation	mm	B12
Precipitation seasonality	–	B15
Precipitation of the Warmest Quarter	mm	B18
Precipitation of the Coldest Quarter	mm	B19
Solar radiation intensity in January	kJ m^−2^ day^−1^	R1
Solar radiation intensity in June	kJ m^−2^ day^−1^	R6
Solar radiation intensity in July	kJ m^−2^ day^−1^	R7
Average wind speed in July	m s^−1^	W7
Average wind speed in August	m s^−1^	W8
Transpiration pressure in February	kPa	P2
Transpiration pressure in July	kPa	P7
Topsoil gravel content	%vol	S1
Topsoil sand fraction	% wt	S2
Topsoil clay fraction	% wt	S3
Topsoil reference bulk density	kg dm^3^	S4
Topsoil organic carbon	% weight	S5
Topsoil pH (H_2_O)	− log(H^+^)	S6
Topsoil salinity	dS m	S7
Subsoil reference bulk density	kg dm^3^	S8
Subsoil CEC	cmol/kg	S9

The topsoil depth is defined as 0–30 cm, and the subsoil depth is defined as 31–100 cm.

### Data processing

ArcGIS 10.2 (ESRI, USA) was used to retrieve environmental factors. Calculations for the differences of SPAD and chlorophyll *a* fluorescence parameter between provenances were done in Excel 2016 (Microsoft Office, USA) and R version 4.0.2 (R Core Team). Data were tested for normality and homogeneity of variance before the analysis and were log-transformed prior to analysis to provide normality when required. Pearson correlation coefficient was calculated using the R. The curves of chlorophyll *a* fluorescence transient were normalized to the interval [0, 1]. Differences between means of provenances were evaluated with the LSD test at 0.05 and 0.01. The coefficient of variation (*CV* = (Standard deviation/mean) × 100%) was calculated in order to investigate the degree of dispersion of φ_Po_, ψ_Eo_, δ_Ro_, and RC/ABS. The relationship between PI_total_ (the mean of the provenances) and provenance’s environmental factor was examined using the Maximal Information Coefficient correlation analysis (MIC). MIC was calculated using the “Minerva” of R package. The relationship between PI_total_ and the warmest quarter precipitation of provenance was determined using regression analyses through the Origin 2018 (Origin Lab Corporation, USA). The interactions among the provenance, test site, and test site:provenance were analyzed by the Generalized Linear Mixed Model (GLMM), calculated using the “nlme” of R package.

### Ethics approval

The experimental research and field studies on plants, including the collection of plant material, complied with relevant institutional, national, and international guidelines and legislation. The appropriate permissions and licenses for the collection of plant or seed specimens were obtained for the study. Prof. Xinping Li identify the plant material. The plant materials were deposited in the herbarium of Shanxi Academy of Forestry and Grassland Sciences, China. The voucher ID of the specimen was from LKY-LZY01 to LKY-LZY10.

## Results

### Relative chlorophyll content analysis

**Figure 2:**
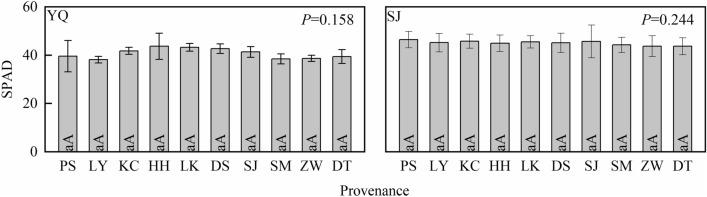
Relative chlorophyll content analysis of *Quercus liaotungensis* seedlings between provenances. YQ represents the Yangqu test site, and SJ represents the Sanjiao test site. Different lowercase letters indicate a significant difference at the 0.05 level, and uppercase letters indicate a significant difference at the 0.01 level.

Differences in SPAD were not significant between the 10 provenances of *Q. liaotungensis* in the two test sites, respectively. SPAD in Yangqu test site ranged from 43.70 to 37.02 with a mean of 40.66 ± 2.22, and SPAD in Sanjiao test site ranged from 47.23 to 42.77 with a mean of 44.86 ± 1.16. It indicated that the material basis for light energy absorption of the tested leaves were similar in the same test site (Fig. 2).

### Chlorophyll *a* fluorescence transient analysis

**Figure 3:**
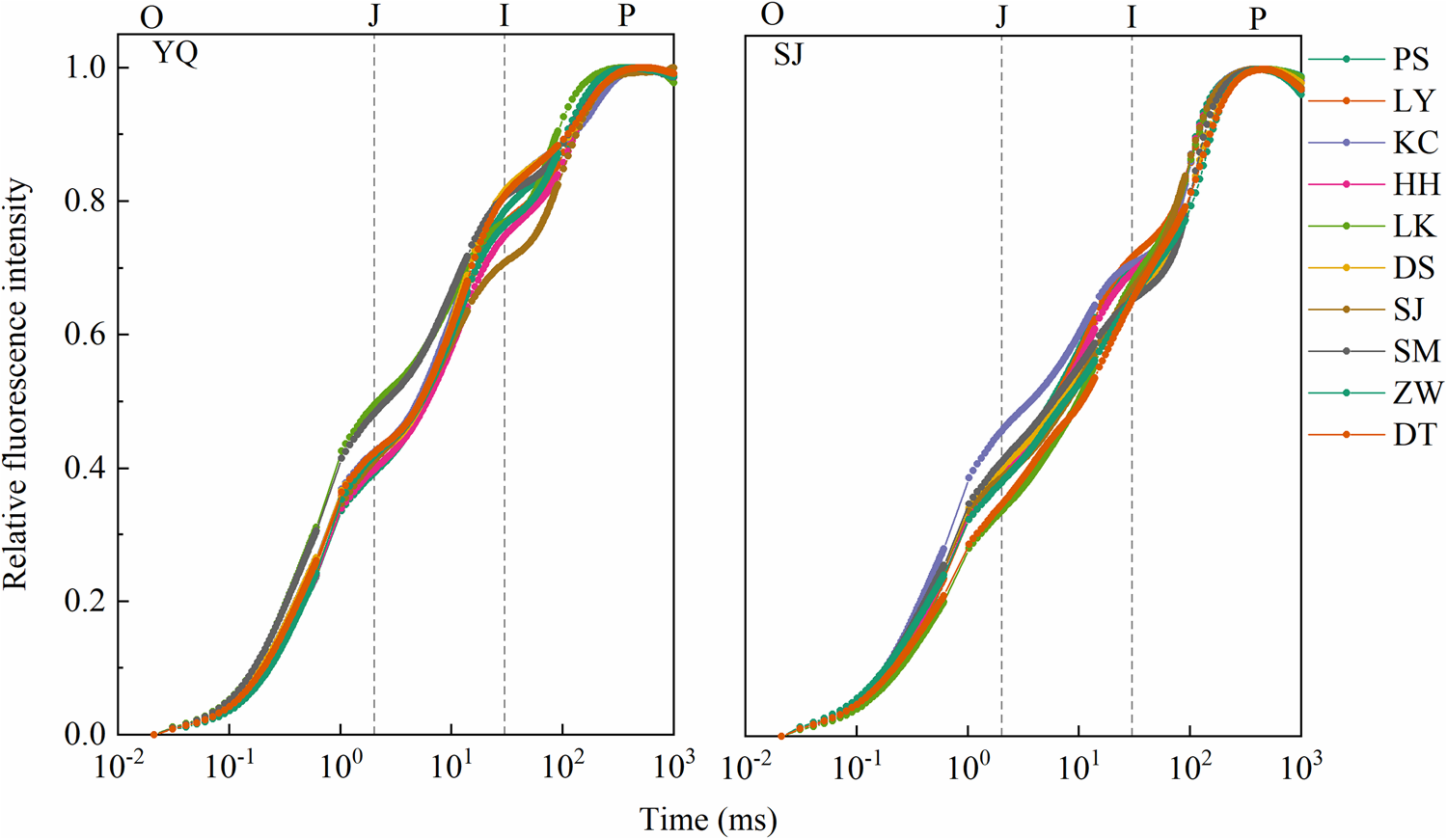
Normalized chlorophyll a fluorescence transient of *Q. liaotungensis* provenances. The O, J, I, and P steps are marked in the figure, where: O is for origin, J and I are for the intermediary fluorescence levels at 2 ms and 30 ms, and *P* is for the peak^20^. YQ represents the Yangqu test site, and SJ represents the Sanjiao test site.

Both normalized chlorophyll *a* fluorescence transient showed the typical OJIP shape in two test sites. Differences in each step's relative fluorescence intensity between provenances were not evident, indicating that the electron transfer status between PSII and PSI of all provenances were similar (Fig. 3).

### Chlorophyll *a* fluorescence parameter analysis

**Figure 4:**
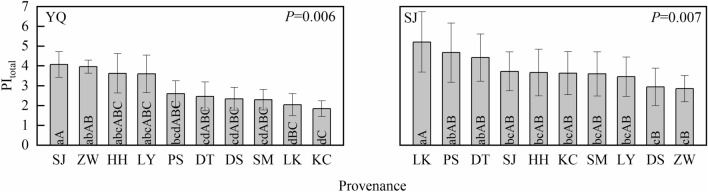
PI_total_ analysis of *Quercus liaotungensis* seedlings between provenances. *P*-value indicates the differential significance of PI_total_ between provenances in the same test site. YQ represents the Yangqu test site, and SJ represents the Sanjiao test site. Different lowercase letters indicate a significant difference at the 0.05 level, and uppercase letters indicate a significant difference at the 0.01 level.

PI_total_ reflected the photochemical activity of the electron transfer chain from the PSII oxygen-evolving complex to the final electron acceptors of PSI. Figure 4 showed that there were significant differences in PI_total_ between provenances in each test site. In Yangqu test site, SJ provenance had the highest PI_total_, with an average value of 4.07, reaching 2.21-fold higher (p < 0.01) than that of the lowest PI_total_ (KC provenance) in the same test site. In Sanjiao test site, the average PI_total_ value of LK provenance was 5.21, reaching 1.83 -fold higher (p < 0.05) than that of the lowest PI_total_ (ZW provenance) in the same test site.

PI_total_ is based on four independent parameters: φ_Po_, ψ_Eo_, δ_Ro_, and RC/ABS. φ_Po_, ψ_Eo_, and δ_Ro_ represent the probabilities that electron is transferred to the Q_A_, PQ, and final PSI acceptor side. RC/ABS represents the density of PSII reaction centers. Table 4 showed the analysis of φ_Po_, ψ_Eo_, δ_Ro_, and RC/ABS of *Q. liaotungensis* seedlings between provenances. The variation analysis (using *CV*) showed the data-sparse in descending order were δ_Ro_, ψ_Eo_, and φ_Po_ between provenances. The differences of δ_Ro_ and ψ_Eo_ between provenances were highly significant (*p* < 0.01) in both test sites. The *CV* of the PSII reaction center was 7.53 (in Yangqu test site) and 7.61 (in Sanjiao test site), implying the data-sparse of RC/ABS between provenances were prominent in both test sites. These results suggest that the difference in probability of electron transfer from PSII to PSI and the number of active reaction centers in PSII were gradually increased between provenances, causing a significant difference in PI_total_ between provenances in the same test site.

**Table 4:** φ_Po_, ψ_Eo_, δ_Ro_ and RC/ABS analysis of *Quercus liaotungensis* seedlings between provenances.

Code	Yangqu test site				Sanjiao test site			
	φ_Po_	ψ_Eo_	δ_Ro_	RC/ABS	φ_Po_	ψ_Eo_	δ_Ro_	RC/ABS
PS	0.82 ± 0.03	0.60 ± 0.02	0.39 ± 0.06	0.58 ± 0.09	0.81 ± 0.02	0.61 ± 0.04	0.51 ± 0.07	0.63 ± 0.03
LY	0.84 ± 0.01	0.59 ± 0.01	0.40 ± 0.01	0.67 ± 0.09	0.80 ± 0.02	0.62 ± 0.03	0.46 ± 0.04	0.58 ± 0.04
KC	0.82 ± 0.03	0.58 ± 0.02	0.33 ± 0.06	0.59 ± 0.05	0.79 ± 0.03	0.55 ± 0.04	0.55 ± 0.10	0.60 ± 0.08
HH	0.83 ± 0.02	0.61 ± 0.02	0.42 ± 0.05	0.64 ± 0.04	0.80 ± 0.01	0.62 ± 0.02	0.49 ± 0.07	0.57 ± 0.03
LK	0.78 ± 0.05	0.51 ± 0.03	0.50 ± 0.08	0.54 ± 0.07	0.81 ± 0.02	0.67 ± 0.03	0.49 ± 0.05	0.61 ± 0.05
DS	0.85 ± 0.01	0.59 ± 0.02	0.32 ± 0.03	0.61 ± 0.05	0.75 ± 0.03	0.57 ± 0.05	0.58 ± 0.05	0.51 ± 0.04
SJ	0.81 ± 0.02	0.60 ± 0.02	0.50 ± 0.06	0.62 ± 0.03	0.78 ± 0.03	0.63 ± 0.04	0.52 ± 0.08	0.54 ± 0.02
SM	0.83 ± 0.01	0.54 ± 0.02	0.40 ± 0.03	0.59 ± 0.03	0.74 ± 0.02	0.60 ± 0.03	0.59 ± 0.05	0.53 ± 0.04
ZW	0.85 ± 0.01	0.59 ± 0.01	0.41 ± 0.02	0.72 ± 0.01	0.74 ± 0.03	0.63 ± 0.03	0.55 ± 0.02	0.50 ± 0.06
DT	0.84 ± 0.01	0.60 ± 0.04	0.33 ± 0.03	0.63 ± 0.03	0.77 ± 0.03	0.66 ± 0.02	0.53 ± 0.03	0.56 ± 0.08
Mean	0.83	0.58	0.40	0.62	0.78	0.62	0.53	0.56
SD	0.02	0.03	0.06	0.05	0.03	0.03	0.04	0.04
*CV* (%)	2.48	5.20	15.14	7.53	3.36	5.68	7.40	7.61
*P-*values	0.104	< 0.001	0.001	0.100	< 0.001	< 0.001	0.001	< 0.001

Mean and SD represent the average value and standard deviation of the parameter, respectively. *CV* and *P*-values represent the coefficient of variation and difference significance between provenances, respectively.

### Environmental factor analysis

**Figure 5:**
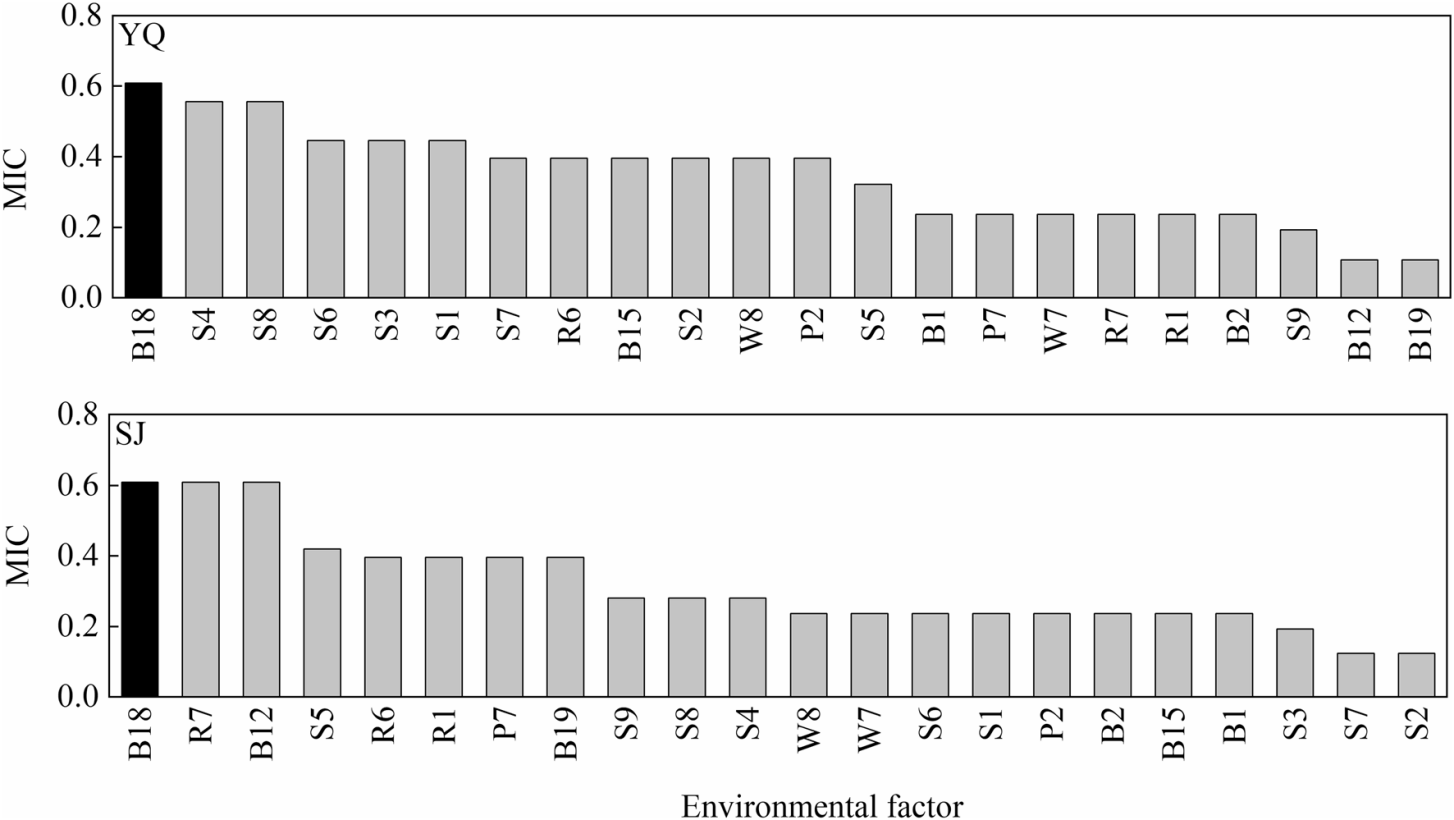
MIC correlation coefficient of *Quercus liaotungensis* seedlings between PI_total_ and environmental factor. YQ represents the Yangqu test site, and SJ represents the Sanjiao test site. See Table 3 for the meaning of environmental factor code. Black bars indicate the same environmental factor with the highest MIC between test sites.

MIC correlation analysis is used to find linear and nonlinear correlations between variables. The MIC value ranges from 0 to 1. The closer the MIC value to 1.0, the higher the correlation of the variable is. As shown in Fig. 5, the warmest quarter precipitation (B18) had the highest MIC value in both test sites. The results showed that the warmest quarter precipitation (B18) in provenance was closely related to the PSII photochemical activity of *Q. liaotungensis* seedlings. Additionally, the solar radiation intensity in July (R7) and the annual precipitation (B12) also significantly affected the PSII photochemical activity in the Sanjiao test site.

**Figure 6:**
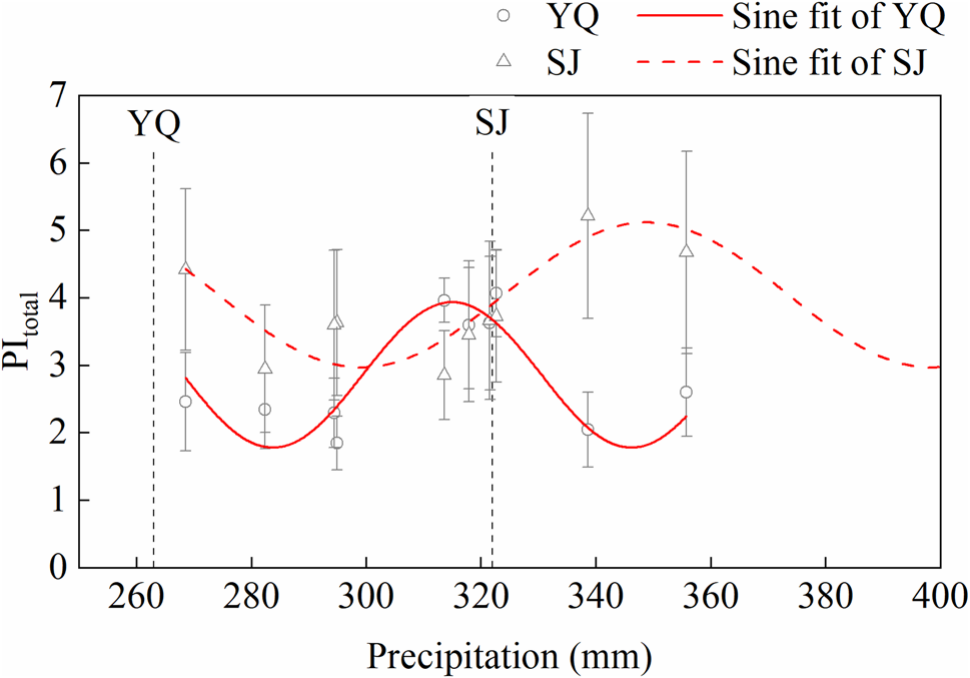
Regression analysis of *Quercus liaotungensis* seedlings between PI_total_ and precipitation of the warmest quarter in provenance. YQ represents the Yangqu test site, and SJ represents the Sanjiao test site. The dotted lines indicate the precipitation of the warmest quarter in two test sites. R^2^ of Sine fit of YQ is 0.90, *P* < 0.01, and R^2^ of Sine fit of SJ is 0.77, *P* < 0.01.

The regression analysis was used to predict the precipitation of the warmest quarter (B18) of provenance and PI_total_ in the two test sites. The results showed that the precipitation of the warmest quarter (B18) and PI_total_ could be fitted by the Sine function in the two test sites (R^2^ was 0.90 in Yangqu test site and was 0.77 in Sanjiao test site). However, the peak location of the fitted curves differed between the two test sites. The fitting curve of Yangqu test site had a peak of around 310 mm, while in Sanjiao test site, the peak was around 350 mm. Compared with the fitted curve of Yangqu, the peak location of the Sanjiao’s fit curve was shifted to the right. Interestingly, Fig. 6 showed that the precipitation of Sanjiao was shifted to the right compared with Yangqu.

### Inter-location PI_total_ difference analysis

#### Comparison of SPAD, chlorophyll *a* fluorescence parameter, and environmental factor

Table 5 showed the influence of provenance and test site on SPAD and chlorophyll *a* fluorescence parameter. Test site had significant effect on parameters and test site:provenance had significant combined effect on φ_Po_ and RC/ABS, while provenance had no effect. For PI_total_, only test site had a significant influence (*P* = 0.04).

**Table 5:** The interaction among provenances, test site, and test site:provenance analysis of *Quercus liaotungensis* seedlings.

Factory	*P*-value					
	SPAD	PI_total_	φ_Po_	ψ_Eo_	δ_Ro_	RC/ABS
Provenance	0.45	0.90	0.20	0.54	0.56	0.41
Test site	< 0.01**	0.04*	< 0.01**	0.06	< 0.01**	0.02*
Test site:Provenance	0.67	0.51	0.02*	0.35	0.37	0.04*

*Significant difference at the 0.05 level, and **significant difference at the 0.01 level.

**Figure 7:**
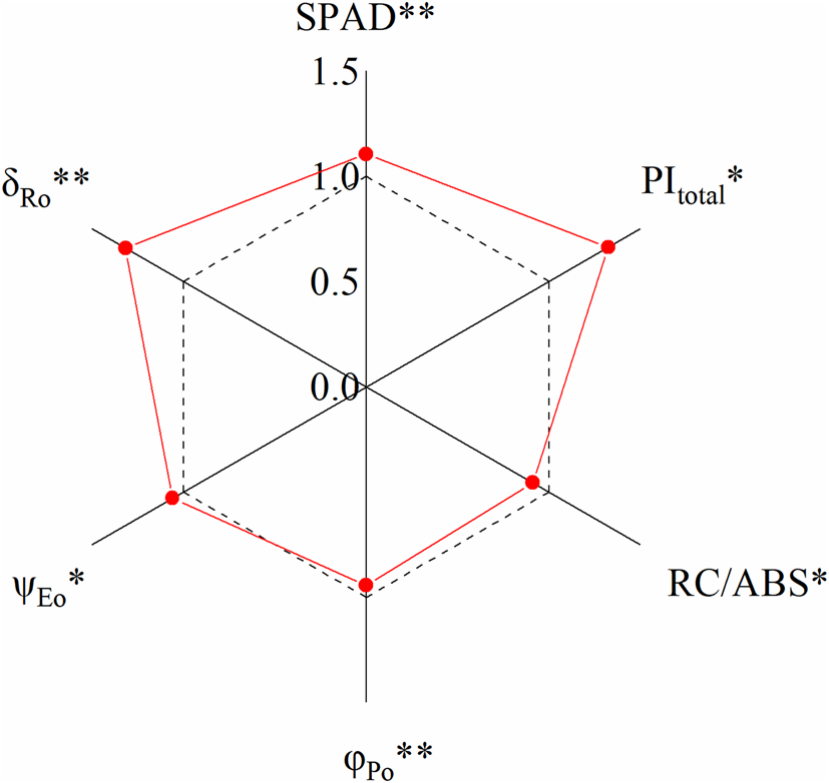
Ratio of the parameter mean value of Sanjiao test site to Yangqu test site. *The parameter is observed significant differences between Yangqu test site and Sanjiao test site at the 0.05 level. **Significant differences at the 0.01 level. The dotted lines indicate the 1.0×.

The average values of SPAD, ψ_Eo_, and δ_Ro_ of all provenances were significantly higher, and the average values of φ_Po_ and RC/ABS were lower in Sanjiao test site compared with Yangqu test site (Fig. 7). These results indicated that SPAD, ψ_Eo_, and δ_Ro_ were the key parameters which caused the difference in PI_total_ between the two test sites.

**Figure 8:**
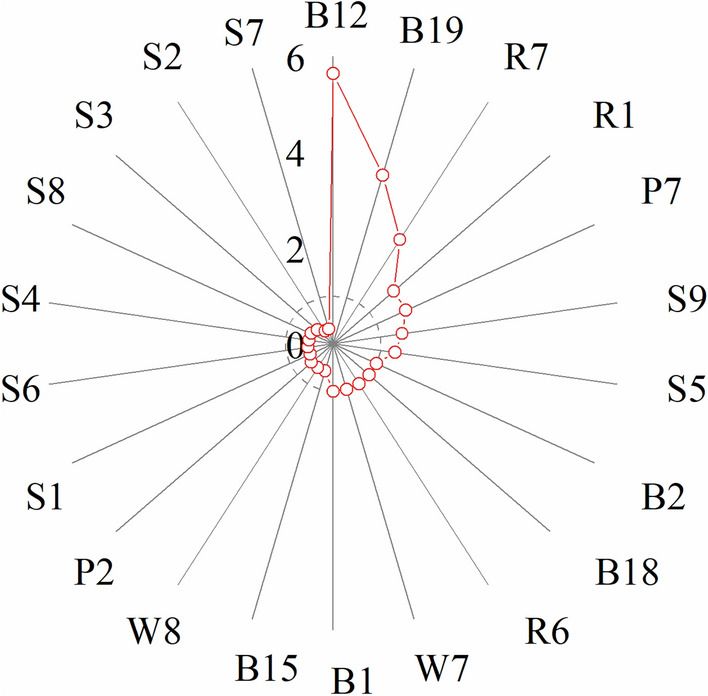
Ratio of Sanjiao test site to Yangqu test site for each environmental factor. The dotted lines indicate the 1.0×.

In Sanjiao test site, the annual precipitation (B12) and the coldest quarter precipitation (B19) were 5.6 and 3.7 times higher than those in Yangqu test site, respectively. Additionally, the topsoil salinity (S7) and the topsoil sand fraction (S2) in Yangqu test site were 3.2 times higher than those in Sanjiao test site (Fig. 8). The result demonstrated precipitation, topsoil salinity, and sand fraction were the main environmental factors that cause differences in PI_total_ between the two test sites.

## Discussion

In the two test sites, there were significant differences in PI_total_ between the 10 provenances of *Q. liaotungensis* when they were grown in the same environment. This finding was similar to the results of the research on interspecies differences in PI_ABS_ of European beech (*Fagus sylvatica*) by Kurjak, et al. ^36^, reflecting the difference in PSII photochemistry activity between provenances. PI_ABS_ is related to only the process of electron transport to the PQ pool^25^. In this study, PI_total_ was used to evaluate the state of the linear photosynthetic electron transfer chain between PSII and PSI. Therefore, the results of this study could be interpreted as the total PSII photochemical activity of *Q. liaotungensis* of different geographic provenance.

From the perspective of electron transfer from PSII to PSI, the *CV* of δ_Ro_ was the highest, and the *P*-value of δ_Ro_ was 0.001 (Table 4). This phenomenon indicated that electron transfer between PQ and PSI electron acceptors of *Q. liaotungensis* appeared to be most sensitive to environmental changes. On the other hand, this phenomenon might indicate that the PSI acceptor side of *Q. liaotungensis*'s structure was relatively unstable and had higher diversity between different provenances. The data-sparse of RC/ABS between provenances of *Q. liaotungensis* was also rather large. Several reports have confirmed that environmental stress can cause decreased PSII active reaction centers^41–43^. In this study, the normalized OJIP curves of provenances did not show obvious signs of stress. Hence, a possible explanation for the data-sparse of RC/ABS is that some provenances encountered mild stress in the test site due to ecological distance (between the original field site and the common garden).

The study results showed that the precipitation of the warmest quarter of provenance location was closely related to the PSII photochemical activity of *Q. liaotungensis* in both test sites. Yangqu and Sanjiao test sites were both characterized by a temperate continental monsoon climate, with high temperatures and plentiful rainfall in the summer. Related studies have found that the net photosynthetic rate of *Q. liaotungensis* reached maximum in mid-July^44^. These results suggest that the PSII photochemistry activity of different *Q. liaotungensis* provenances was closely related to the water supply during the growing season. This speculation is similar to the conclusion of Wu et al. that sufficient water supply during the growing season can significantly increase the carbon assimilation rate of plant^45^.

The precipitation—PI_total_ fitted curves first raised and then fell in the two test sites. Studies have confirmed that drought stress^46,47^ and flooding stress^48,49^ will significantly reduce the photochemical activity of PSII. Ecological distance might lead to excess or lack of water for some provenance. As a result, the curves first raised and then fell. Furthermore, the peak location of the Sanjiao’s fitted curve was shifted to the right compared with the Yangqu’s curve. GLMM analysis revealed a significant effect of the test site on PI_total_ (Table 4) and the Yangqu test site with lower precipitation in the warmest quarter than the Sanjiao test site (Fig. 8). Based on this hypothesis, precipitation can be considered as the main cause of this phenomenon, and more provenances may suffer from mild drought stress in the Yangqu test site.

PI_total_ and the peak location of the precipitation—PI_total_ fitted curves were varied between the two test sites. As shown in Fig. 7, the internal factors causing this phenomenon were the difference in leaf chlorophyll content and the probability of electron transfer to PQ and final PSI acceptors. Because photosynthetic pigment play an essential role in absorption and transfer of light energy^50^, and φ_Po_, ψ_Eo_, δ_Ro_, and RC/ABS were key indicators of the total PSII photochemical activity. However, other internal factors such as morphological and physiological traits may also affect the PSII photochemical activity between provenances^37^. Precipitation and soil conductivity have significant effects on PSII photochemical activity of plant leaves^51^. Figure 8 also showed that the external factors resulting in this phenomenon are the different precipitation and upper soil conductivity in the two test sites.

## Conclusion

We have shown that the differences in PI_total_ of *Q. liaotungensis* seedlings between provenances do exist, and the different trends of PI_total_ can be fitted by the Sine function in the two test sites. These results helped to screen the provenance of *Q. liaotungensis* with high and stable photosynthetic efficiency by using PI_total_. In the future, it is necessary to eliminate genetic differences within provenance, increase the number of provenances, and incorporate more environmental factors to improve the accuracy of the OJIP-test.
